# Planetary health diet with einkorn as a potential preventive strategy to improve interdental microbiota, oral health and quality of life: a pilot clinical trial

**DOI:** 10.1080/20002297.2026.2626138

**Published:** 2026-02-09

**Authors:** Audrey Murat-Ringot, Laurie Fraticelli, Denis Bourgeois, Lama Basbous, Anne Lastmann, Laurence Mayaud, Marie-Thérèse Charreyre, Romain Lan, Florence Carrouel

**Affiliations:** a Laboratory ‘Health, Systemic, Process’ (P2S), UR4129, University Claude Bernard Lyon 1, University of Lyon, Lyon, France; b Hospices Civils de Lyon, Lyon, France; c Universite Claude Bernard Lyon 1, INSA Lyon, Université Jean Monnet, CNRS UMR 5223, Ingénierie des Matériaux Polymères, Villeurbanne Cedex, France; d Laboratory ADES, Aix Marseille University, CNRS, EFS, Marseille, France

**Keywords:** Sustainable diets, microbiota, oral health, health promotion, planetary health diet, einkorn wheat, global health

## Abstract

**Background:**

Non-communicable diseases (NCDs) involve chronic inflammatory mechanisms to which periodontal periodontal dysbiosis may contribute. The Planetary Health Diet (PHD), a sustainable plant-based model for NCD prevention remains poorly explored regarding its impact on the interdental microbiota.

**Objective:**

To evaluate whether an einkorn-enriched PHD can reduce interdental microbiota dysbiosis and promote oral and general health.

**Design:**

BIOQUALIM was a 3-month, single-arm, uncontrolled longitudinal pilot trial conducted under real-life conditions. Participants reduced their meat consumption by 50% and consumed  ≥100g/day of whole-grain einkorn. Interdental microbiota, periodontal indices, digestive comfort, and quality of life were assessed at baseline, one and three months.

**Results:**

Total bacterial load (*p* = 0.0236), *Tannerella forsythia* (*p* < 0.001) and *Treponema denticola* (*p* < 0.001) decreased significantly. Periodontal health improved significantly—probing depth decreased from 1.14 ± 0.70 mm to 0.58 ± 0.51 mm (*p* < 0.0001) and clinical attachment loss from 1.33 ± 0.73 mm to 0.60 ± 0.57 mm (*p* < 0.0001). Gingival inflammation decreased by > 60%, and bleeding on probing decreased by >70% (*p* = 0.0004). Digestive comfort improved— normalized stool frequency (*p* = 0.0009) and reduced postprandial drowsiness (*p* = 0.0196). Quality-of-life scores increased significantly (*p* = 0.039).

**Conclusion:**

An einkorn-enriched PHD was associated with improved interdental microbiota, periodontal health, and well-being, supporting a One Health link between sustainable nutrition, oral microbiota modulation and inflammation-related pathways.

## Introduction

Non-communicable diseases (NCDs) such as cardiovascular diseases, cancer, diabetes, and periodontal conditions account for nearly 75% of global deaths, representing over 43 million deaths annually [1]. These pathologies are strongly influenced by environmental and behavioural factors, among which diet plays a central role [2]. Within this perspective, the One Health concept emphasises the interdependence of human, animal, and environmental health, calling for transdisciplinary approaches to address global challenges [3]. Among NCD-related conditions, periodontal disease represents a particularly relevant and modifiable factor, as it shares common inflammatory pathways with systemic diseases and is directly influenced by behavioural and dietary factors [4]. The Planetary Health Diet (PHD), proposed by the EAT-Lancet Commission, aims to prevent NCD mortality while lowering greenhouse gas emissions through a predominantly plant-based dietary model [5,6].

Shifting from animal to plant proteins requires diversification of plant-based protein sources, particularly legumes and cereals. However, modern agricultural practices and biodiversity loss have reduced the nutritional quality of commonly consumed species [7]. Among cereals, ‘ancient wheat species’, such as einkorn (*Triticum monococcum ssp. monococcum*), contain high levels of micronutrients and bioactive compounds with antioxidant and anti-inflammatory properties [8]. For instance, einkorn provides up to 25% more phytosterols and three to eight-fold more lutein (a carotenoid) than modern wheat varieties, micronutrients associated with reduced oxidative stress and systemic inflammation [9]. Although the concentration of these bioactive compounds may vary substantially according to genotype and environment [10,11], and although their bioavailability and clinical relevance in real-life dietary settings remain incompletely established [9,12], these features suggest a preventive potential against NCDs.

Beyond systemic effects, diet also has a direct influence on oral microbiome [4]. Interdental microbiota dysbiosis, characterised by the overgrowth of pathogens from the red or orange complex of Socransky such as *Porphyromonas gingivalis*, *Treponema denticola* or *Tannerella forsythia*, is a major risk factor for periodontal disease, associated with systemic comorbidities [2,4,13]. These bacterial complexes are consistently recognised as the species most strongly associated with periodontal inflammation and disease progression, and several of these pathogens have also been identified as biological risk markers in systemic inflammatory and NCDs [2,4,13]. Conversely, diets rich in plant proteins and fibres may favourably modulate oral microbial communities, thereby reducing gingival inflammation, including bleeding on probing (BoP) [14,15]. To date, no clinical studies have directly assessed whether a sustainable dietary model such as the PHD can influence interdental dysbiosis or periodontal parameters. Accordingly, focusing on these red and orange complex pathogens provides a clinically and biologically relevant framework to explore potential diet-related modulation at the oral–systemic interface.

Within this broader One Health and Planetary Health framework, this pilot study aimed to generate proof-of-concept signals linking a PHD-like dietary pattern enriched with an ancient wheat (einkorn) to interdental microbiota dysbiosis (operationally defined as the increased burden and longitudinal changes of selected periodontal pathogens from the red and orange complexes of Socransky), periodontal clinical indices, oral and general health, and quality of life. The hypothesis was that adherence to PHD enriched with einkorn would be associated with favourable changes in interdental microbiota composition, periodontal clinical parameters, general health indicators, and quality of life over time. This would provide a proof-of-concept for linking sustainable nutrition with oral health within a ‘One Health’ framework.

## Materials and methods

### Study design and setting

BIOQUALIM clinical study was a single-arm longitudinal pilot clinical trial, conducted between January and June 2025 at the Civil Hospices of Lyon (HCL, Lyon, France) [16]. It was designed as an exploratory, hypothesis-generating pilot and was not intended to allow causal inference. The study followed the TREND statement [17] and STROBE guidelines [18]. The trial was registered at ClinicalTrials.gov (NCT06315088).

### Participants

Eligible participants were men and women, aged 20–60 years, with an omnivorous diet and body mass index (BMI) ≥ 18 kg/m², who provided written informed consent.

Exclusion criteria were: (i) active smoking, (ii) food allergies or intolerances, (iii) inability to consume dairy or solid products, (iv) high risk of infective endocarditis, (v) chronic systemic diseases, (vi) antibiotic use in the month before enrolment, (vii) pregnancy or breastfeeding, (viii) psychiatric care or legal protection status, (ix) fewer than 20 natural teeth, (x) untreated caries, (xi) regular interdental hygiene practices, (xii) orthodontic appliances, (xiii) current use of mouthrinses and (xii) periodontal disease (periodontal lesions stage ≥ II according to the 2017 Chicago classification [19] (i.e. PD ≥ 4 mm, and/or CAL ≥ 4 mm) and/or generalised (>30% of sites)).

### Sample methodology

Recruitment was performed, 25th November 2024 to 25th February 2025, via (i) a database of volunteers from the P2S laboratory, (ii) internal calls within the HCL, and (iii) social media posts by the P2S research team.

### Intervention

Participants were instructed to follow a three-month dietary programme (February 2025 to May 2025) consisting of reduction of meat intake by 50%, compared to their usual diet and daily consumption of einkorn (≥100 g/day, cooked whole grains, 6 days per week). They did not receive any professional prophylaxis before or during the study, were not given specific toothbrushing or interdental hygiene instructions and were not allowed to modify their usual oral hygiene habits during the study period.

The einkorn, provided to each participant, was organically cultivated in the Lyon region, harvested during summer 2024 from a single plot of land to ensure homogeneity. To support adherence, four culinary workshops were organised providing recipes and practical guidance on incorporating einkorn into meals. Each two-hour workshop included the preparation of one sweet and one savoury recipe, both incorporating whole grains einkorn. The recipes varied for each workshop to showcase diverse options. Participants received printed copies at the end to encourage continued exploration of plant-based proteins in their diets.

### Clinical, microbiological, and questionnaire assessments

Periodontal evaluations were performed at baseline (T0), 1 month (T1), and 3 months (T2) by a single trained and calibrated periodontist, ensuring consistency of measurements throughout the study. Intra-examiner calibration was performed prior to study initiation to ensure reproducibility of probing depth and clinical attachment level measurements. Due to the nature of the dietary intervention, blinding of the examiner to study time points was not feasible; however, standardised examination procedures were strictly applied at each visit. The following clinical parameters were assessed on all natural teeth present at the time of examination, excluding third molars:

- BoP [20,21] was assessed using a dichotomous scoring system, recording the presence or absence of bleeding within 30 seconds following probing (0 = no bleeding; 1 = bleeding present). Measurements were performed at four sites per tooth (mesiobuccal, distobuccal, mesiopalatine, and distopalatine).
- Gingival inflammation was assessed using the gingival index (GI) [22] on a four-point ordinal scale ranging from 0 to 3, based on visual inspection. Scores reflected increasing severity of inflammation, from absence of clinical signs (score 0), through mild changes in colour and texture (score 1), to moderate inflammation with erythema, oedema, and bleeding on probing (score 2), and severe inflammation with pronounced redness, tissue enlargement, and a tendency to spontaneous bleeding (score 3). The gingivitis score was calculated as the mean GI across all examined sites.
- Plaque index (PIE) was evaluated using the Rustogi-modified Navy Plaque Index [23]. For each tooth, plaque presence was recorded on buccal and lingual surfaces subdivided into nine areas each, using a dichotomous scoring system (0 = absence; 1 = presence). This approach yields 18 scored sites per tooth and enables detailed assessment of plaque distribution at marginal and interproximal levels, as well as at the whole-mouth scale.
- Probing depth (PD) [24] was measured as the distance, expressed in millimetres, between the gingival margin and the base of the periodontal sulcus or pocket. Measurements were obtained at four sites per tooth (mesiobuccal, distobuccal, mesiopalatine, and distopalatine) using a calibrated periodontal probe.
- Clinical attachment loss (CAL) [21] was determined by combining probing pocket depth with gingival recession, defined as the distance between the cemento-enamel junction and the base of the periodontal sulcus or pocket. CAL measurements were recorded at four sites per tooth (mesiobuccal, distobuccal, mesiopalatine, and distopalatine).

For each of these clinical parameters, site-specific measurements were first averaged at the tooth level, and participant-level scores were subsequently calculated as the mean of all tooth-level values.

General clinical parameters—weight, abdominal perimeter, and blood pressure parameters (systolic blood pressure (SBP) and diastolic blood pressure (DBP))—were also recorded at these time points.

The microbiological analysis targeted red and orange complex periodontal pathogens for their established clinical relevance, enabling quantitative monitoring of pathogen burden in the interdental biofilm in this pilot study, rather than characterisation of global microbial diversity. A targeted quantitative PCR approach previously described and validated for interdental biofilm analysis [25] was chosen to ensure analytical sensitivity, reproducibility, and longitudinal comparability in this exploratory pilot study, focusing on clinically relevant periodontal pathogens rather than community-wide microbiome profiling. Therefore, interdental biofilm was collected from four standardised sites (15–16, 25–26, 35–36, 45–46) at T0, T1, and T2. When a tooth was missing, the adjacent medial site was sampled. Appropriate interdental brush sizes (IDBs, Curaden, Kriens, Switzerland) were selected during clinical assessment. Target teeth were isolated with sterile cotton rolls, and biofilm was collected using a sterile, calibrated IDB. Four IDBs per participant were pooled into a single sterile 1.5 mL tube and stored at −20 °C until processing. DNA was extracted using the QIAcube HT system with the Cador Pathogen 96 Kit (Qiagen, Hilden, Germany) following manufacturer instructions. DNA quality and concentration were assessed via absorbance at 260/280 nm, and all samples were normalised to 50 ng/µL. Two microliters of DNA were used for quantitative real-time PCR to quantify total bacterial load and bacteria from the red complex (*P. gingivalis, T. forsythia, T. denticola*) and orange complex (*Prevotella intermedia (P. intermedia), Parvimonas micra (P. micra), Fusobacterium nucleatum (F. nucleatum), Campylobacter rectus (C. rectus)*). All assays were performed using species-specific primers previously validated in interdental biofilm studies [25]. All qPCR reactions were performed in duplicates, and mean values were retained for analysis. Negative controls and standard curves were included in each run to verify assay specificity and amplification efficiency. Bacterial quantities were reported as absolute genome copy numbers per pooled interdental biofilm sample (four standardised interdental sites per participant), as determined by quantitative PCR. Limits of quantification (LOQ) for each bacterial target were defined according to published validation data and ranged from 4 to 200 genome copies depending on the species. All procedures were performed by technicians according to a blinded procedure.

Participants completed the MOS-SF36 questionnaire [26,27] at T0, T1, and T2 to assess quality of life, and a survey related to eating habits and digestive comfort at T0 and T2 (supplementary file 1).

Participants with no follow-up data after baseline or at the final visit were considered lost to follow-up (attrition rate).

### Outcomes

The primary outcome was to determine the change in interdental microbiota composition between baseline (T0) and three months (T2), assessed by quantitative PCR (qPCR) for total bacteria and seven periodontal pathogens: three from the red complex of Socransky (*P. gingivalis, T. forsythia* and *T. denticola*) and four from the orange complex of Socransky (*C. rectus, P. micra, P. intermedia* and *F. nucleatum*).

The secondary outcomes were:

(i) to compare the total bacterial load and periodontal pathogens of the interdental microbiota between T0 and T1;
(ii) to evaluate periodontal and oral clinical parameters at T0, T1, and T2;
(iii) to assess general health indicators at T0, T1, and T2;
(iv) to assess quality of life at T0, T1, and T2.

### Sample size

The study was designed as a proof-of-concept pilot. Based on prior evidence [28], achieving 90% power to detect a 0.5 log10 decrease in pathogenic bacterial load with a two-sided *α* of 0.05 required 36 participants. Accounting for an expected 10% attrition rate, the planned recruitment was set at 40 individuals.

### Statistical analysis

Statistical analysis was based on the subjects with a complete participation in the study period. All data were completed; there was no missing value. For microbial outcomes, due to highly skewed distributions and the presence of zero counts for several species, bacterial genome copy numbers were log10(x + 1) transformed prior to analysis. Quantity was expressed as means ± standard deviations. Continuous variables associated with oral and general health were also expressed as means ± standard deviations.

The variables were compared using Wilcoxon signed-rank tests for paired samples (T0 vs T1, T1 vs T2 and T0 vs T2). For quality-of-life outcomes reported in the questionnaires at T0 and T2, the McNemar–Bowker’s test was used to compare the proportions of evolution in the paired samples. Post-hoc pairwise comparisons were conducted with adjustment for multiple testing using the Benjamini–Hochberg false discovery rate (FDR). A two-sided *p*-value < 0.05 was considered statistically significant. Analyses were performed using R (version 4.5.1.).

### Ethical considerations

The study was approved by the Committee for the Protection of Individuals South Mediterranean IV (No. 2024-A00357-40) and complied with the Declaration of Helsinki. All participants provided written informed consent.

## Results

### Characteristics of participants

A total of 27 participants were enroled in the study, of whom 25 completed the 3-month follow-up (two participants withdrew consent after the first month, attrition rate 7.4%) (Supplementary Figure 1). At baseline, the study population presented a median age of 47.0 [37.0-53.0], and 64% of participants were women. All participants reported an omnivorous diet before enrolment. None of the participants had periodontitis.

### Engagement rate to the dietary intervention

Engagement was high, with 92.6% (25/27) of participants declaring they had adhered to the intervention. Adherence was evaluated using structured self-reported questionnaires on dietary habits and frequency of einkorn consumption collected at baseline and at the end of the intervention; participation in the culinary workshops and reported dietary changes provided additional qualitative support for adherence. No objective dietary records, weighing of provided grains, or biological markers of adherence were collected.

Reported consumption (Supplementary Table 1) indicated a significant reduction in red meat (*p* = 0.0045), white meat (*p* = 0.0075), and cured meat (*p* = 0.033), while fish/seafood (*p* = 0.0046) and leguminous intake (*p* = 0.0075) increased. Egg and whole-grain consumption remained stable. No adverse events were reported.

### Evolution of interdental microbiota

#### Evolution of interdental microbiota at group level

Table 1 presents the evolution of total bacterial load as well as bacteria from Socransky’s red and orange complexes over time. Total bacterial load significantly decreased at T1 compared to baseline (T0, *p* = 0.0236) but no significant changes were observed at T2 (*p* = 0.5602), indicating a transient reduction with a rebound toward baseline levels at three months. These group-level changes represent quantitative shifts in the abundance of selected taxa and do not capture functional activity, virulence expression, or community-wide microbial dynamics.

At the species level, all three red complex bacteria decreased from T0 to T2. *T. forsythia* and *T. denticola* showed significant reductions at T1 (*p* < 0.0001 and *p* = 0.0036, respectively), with non-significant decreases at T2, while *P. gingivalis* declined not significantly at both T1 and T2. Within the orange complex, heterogeneous patterns emerged. *P. micra* showed a tendency to decrease between T0 and T1, reaching a significant reduction only at T2 (*p* = 0.0403). Conversely, *P. intermedia* exhibited a significant increase at T1 compared with T0 (*p* = 0.0067), although not maintained at T2. *F. nucleatum* remained stable at all times, while *C. rectus* showed a significant increase at T2 compared with T0 (*p* = 0.0187).

**Table 1.** Changes in total bacterial load and red and orange complex species over time.

	T0 *N* = 25	T1 *N* = 25	T2 *N* = 25	*p*-value^*^ T0-T1	*p*-value^**^ T0-T1	*p*-value T1-T2	*p*-value** T1-T2	*p*-value* T0-T2	*p*-value**T0-T2	Median change T0-T2 (IQR)
Total quantity	23.47 ± 0.66	23.23 ± 0.65	23.42 ± 0.56	**0.0236**	**0.0472**	0.2635	0.4216	0.5602	0.6639	-0.19 (-0.41 to 0.43)
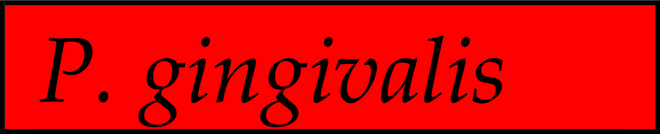	12.01 ± 8.13	11.69 ± 8.43	10.39 ± 9.63	0.8083	0.8083	0.9479	0.9479	0.6639	0.6639	0.00 (-13.13 to 0.61)
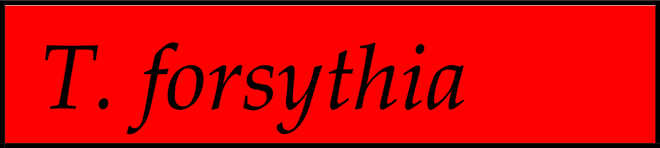	16.83 ± 1.04	15.58 ± 3.45	16.43 ± 1.22	**<0.0001**	0.0008	0.1908	0.4198	0.0587	0.1565	-0.33 (-0.94 to 0.15)
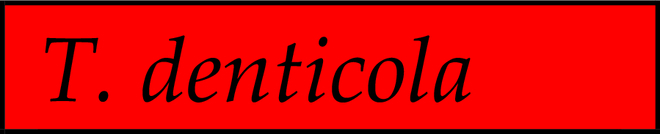	17.15 ± 5.35	15.32 ± 6.14	17.03 ± 5.32	**0.0036**	**0.0144**	**0.0014**	**0.0112**	0.4202	0.6639	-0.09 (-0.73 to 0.47)
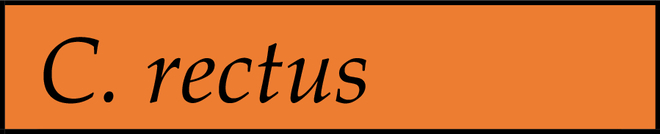	16.26 ± 1.69	16.68 ± 1.51	17.11 ± 1.40	0.1645	0.2193	0.1908	0.4198	**0.0187**	**0.1512**	0.61 (-0.12 to 1.40)
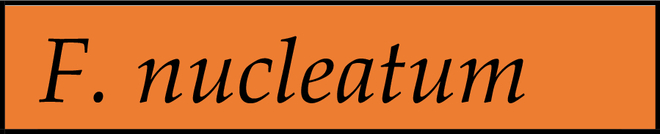	16.76 ± 3.77	17.29 ± 1.20	17.37 ± 1.41	0.4418	0.5049	0.5249	0.5998	0.6338	0.6639	-0.14 (-0.49 to 0.42)
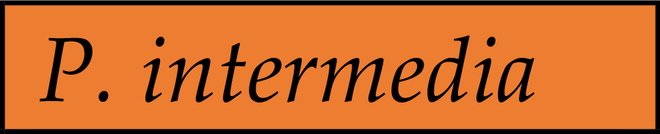	19.05 ± 1.21	19.66 ± 1.04	19.37 ± 1.22	**0.0067**	**0.0178**	0.2099	0.4198	0.2411	0.4822	0.04 (-0.40 to 1.29)
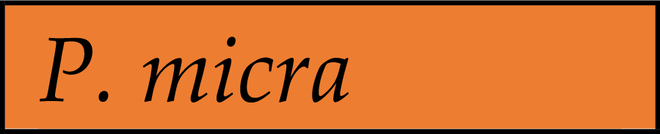	14.21 ± 7.44	10.96 ± 8.47	8.33 ± 9.07	0.0654	0.1046	0.4210	0.5613	**0.0403**	0.1565	-0.57 (-17.00 to 0.86)

Data are expressed as mean ± SD of log10-transformed absolute bacterial genome copy numbers per pooled interdental biofilm sample (log10[x + 1]). Genome copies correspond to total bacterial DNA recovered from four standardised interdental sites per participant. The x + 1 transformation was applied to allow inclusion of zero or below-limit-of-quantification values. Large dispersions reflect marked inter-individual variability in baseline pathogen burden. Observed changes reflect abundance shifts of selected taxa and do not capture functional activity, virulence potential, or community-wide microbial dynamics. The colours refer to (i) the colours of the Socransky complexes for the purple, green, yellow, orange, and red colours.

* Wilcoxon test on paired data.

** Benjamini–Hochberg false discovery rate (Adjusted p-value).

#### Evolution of interdental microbiota at individual level

**Figure 1.**
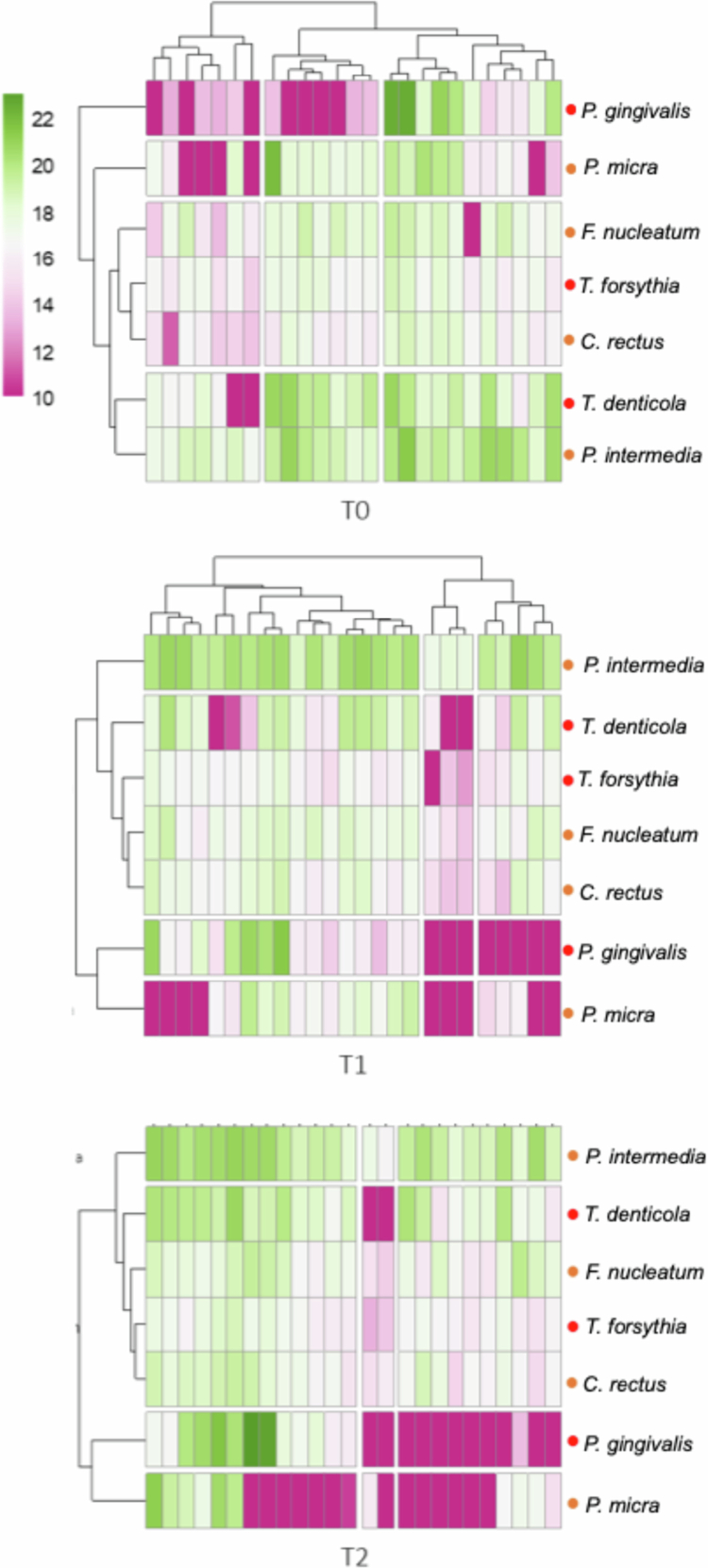
Heatmap of longitudinal follow-up of the log10-transformed bacterial counts. The colours are related to the quantity of bacteria expressed in log10 and range from purple (10 = lowest quantity) to green (22 = highest quantity).

The heatmaps of longitudinal follow-up of the bacterial counts (Figure 1) revealed marked inter-individual heterogeneity, with most participants showing reductions for multiple pathogens, while a minority exhibited increases for selected species.

At the individual level, most changes were observed at one month (T1) and were not consistently maintained at three months (T2) (Figure 1 and Supplementary Table 2), highlighting the transient nature of the observed microbiological effects. Most participants showed reductions for multiple pathogens at one month, while a minority exhibited selective increases, particularly for *P. intermedia* and *C. rectus*. Quantitatively, total bacterial load decreased for 18/25 participants at T1, but this effect was less consistent at T2 (16/25 decreased, 9/25 increased).

Within the red complex, *T. forsythia* decreased for 84.0% (21/25) participants at T1 and 68.0% (17/25) at T2, while *T. denticola* decreased for 63.0% (16/25) at T1 and 52.0% (13/25) at T2. In contrast, *P. gingivalis* displayed highly heterogeneous and bidirectional responses (11 decreased vs. 9–10 increased at both time points).

Among orange complex species, divergent and compensatory patterns were observed. *P. micra* decreased for 56.0% (14/25) participants at both T1 and T2, whereas *C. rectus* increased for 60.0% (15/25) at T1 and 63.0% (16/25) at T2. *F. nucleatum* tended to rise (63.0% (16/25) at T1, 56.0% (14/25) at T2), while *P. intermedia* showed mixed responses, with transient increases for some participants.

### Evolution of oral parameters

Periodontal parameters improved during the intervention (Table 2). PD and CAL decreased by nearly 45–50% at T2 (*p* < 0.0001), while gingivitis and bleeding scores dropped by more than 70% at T1 (p ≤ 0.0001) and remained significantly lower at T2 (*p* = 0.0004). Plaque indices also declined.

**Table 2.** Changes in periodontal clinical parameters and in general health indicators between baseline (T0), 1 month (T1), and 3 months (T2).

	T0 *n* = 25	T1 *n* = 25	T2 *n* = 25	*p*-value* T0-T1	*p*-value** T0-T1	*p*-value* T1-T2	*p*-value** T1-T2	*p*-value* T0-T2	*p*-value** T0-T2	Median change T0-T2 (IQR)
**Probing depth (mm)**	1.14 ± 0.70	1.13 ± 0.73	0.58 ± 0.51	0.1353	0.2029	**<0.0001**	**0.0004**	**<0.0001**	**0.0004**	−0.49 (−0.97; −0.14)
**Clinical attachment loss (mm)**	1.33 ± 0.73	1.28 ± 0.76	0.60 ± 0.57	0.2237	**0.2624**	**<0.0001**	**0.0004**	**<0.0001**	**0.0004**	−0.62 (−1.10; −0.22)
**Plaque index**	0.04 ± 0.08	0.02 ± 0.04	0.02 ± 0.03	**0.0120**	0.0360	0.8129	0.8129	0.2322	0.3483	0.00 (0.00; 0.00)
**Gingival index**	0.36 ± 0.38	0.14 ± 0.31	0.15 ± 0.19	**0.0001**	0.0004	0.7275	0.8129	**0.0004**	**0.0009**	−0.16 (−0.31; −0.04)
**Bleeding on Probing score**	0.19 ± 0.15	0.04 ± 0.05	0.06 ± 0.09	**<0.0001**	0.0004	0.2959	0.6657	**0.0004**	**0.0009**	−0.12 (−0.21; −0.04)
**Height (cm)**	169 ± 10.45									
**Weight (Kg)**	69.97 ± 13.34	69.48 ± 13.64	69.76 ± 13.33	**0.0315**	0.0708	0.4037	0.7266	0.5955	0.6699	0.00 (−1.00; 1.00)
**Abdominal perimeter (cm)**	84.68 ± 10.70	83.0 ± 11.81	86.84 ± 10.94	0.1298	0.2029	**0.0381**	0.1143	0.1368	0.2462	3.00 (−2.00: 7.00)
**Systolic blood pressure (mmHg)**	119.69 ± 12.11	116.16 ± 14.04	117.16 ± 12.10	0.2624	0.2624	0.7531	0.8129	0.6947	0.6947	−1.00 (−8.00: 4.00)
**Diastolic blood pressure (mmHg)**	77.86 ± 8.18	74.24 ± 11.00	75.28 ± 13.04	0.2506	0.2624	0.6259	0.8129	0.5731	0.6699	−1.00 (−10.00; 5.00)

* Wilcoxon test on paired data.

** Benjamini–Hochberg false discovery rate (Adjusted p-value).

### Evolution of general health indicators, digestive comfort and quality of life

Regarding general health indicators, there were no significant changes in body mass index (BMI), abdominal perimeter, blood pressure between baseline and 3 months (Table 2). These parameters remained stable throughout the intervention.

**Figure 2.**
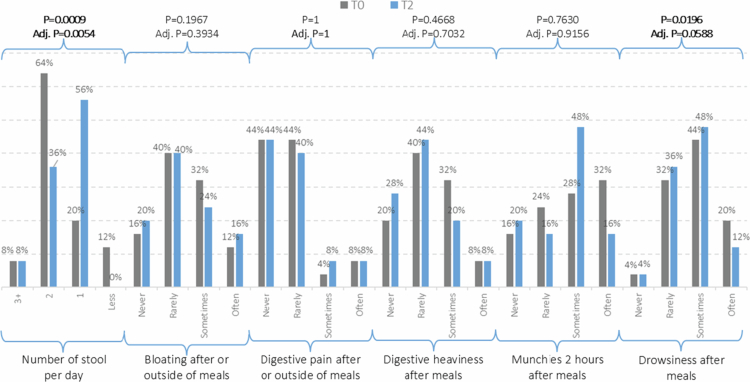
Changes in digestive comfort parameters between baseline (T0) and 3 months (T2).

Regarding the perceived impact on digestive comfort (Figure 2), most self-reported digestive parameters remained unchanged over the 3-month intervention. Bloating, digestive pain, and heaviness after meals, and munchies showed no significant changes over time. For these symptoms, the majority of participants consistently reported ‘rarely’ or ‘sometimes’, with only minor shifts in category distribution.

Among the assessed parameters, only two showed statistically significant changes over time. Stool frequency improved significantly (*p* = 0.0009). At baseline, 12% of participants reported fewer than one bowel movement per day; at T2, none remained in this category. In parallel, the proportion with one stool per day rose from 20% to 56%, indicating a normalisation of intestinal transit.

Drowsiness after meals also significantly decreased (*p* = 0.0196). At baseline, 20% of participants reported being often drowsy after meals, compared to only 12% at T2. Meanwhile, reports of ‘rarely’ and ‘sometimes’ increased slightly, suggesting a shift toward milder, less frequent symptoms.

Regarding the perceived impact on quality of life, self-reported SF-36 quality of life scores showed a slight, non-significant increase at T1, rising from 77.0 [68.0–88.0] at T0 to 79.0 [73.5–83.0] (*p* = 0.2732). At T2, scores increased further to 82.0 [76.0–86.0], reaching statistical significance compared with baseline (*p* = 0.0393). However, the magnitude of this change was modest and occurred in participants who already exhibited relatively high baseline quality of life scores. Overall, these results reflect a modest but consistent improvement in perceived quality of life over the course of the intervention.

## Discussion

To our knowledge, this study represents one of the first interventional clinical trials to evaluate the impact of an einkorn-enriched PHD on interdental microbiota, periodontal health, and quality-of-life outcomes. Previous evidence has been mainly observational. Yue et al. (2025) showed that women with higher diet quality scores had oral microbiota enriched in health-associated taxa [29]. Similarly, Nath et al. (2025) reported that dietary factors and oral hygiene history influenced microbiome composition in healthy adults [30]. Shen et al. (2024) further linked better diet quality to larger oral microbial diversity and reduced mortality in the general population [31]. Our study extends these findings by providing longitudinal interventional trial with quantitative data, specifically targeting interdental dysbiosis, a niche strongly implicated in periodontal diseases [32]. Marked inter-individual heterogeneity in microbiological and clinical responses emerged as a central finding of this study. Changes in interdental pathogen burden were highly variable across participants, underscoring the ecological complexity of the interdental space and indicating that responses to the dietary intervention were not uniform. Such variability, illustrated by the longitudinal heatmap of individual microbiota profiles (Figure 1), suggests that dietary modulation of the interdental microbiota may depend on individual baseline profiles, host-related factors, and microbial resilience, supporting a precision-nutrition rather than a generalised prevention framework [33].

The significant decreases in *T. forsythia* and *T. denticola* are consistent with a potential association between adherence to an einkorn-enriched PHD and changes in key red complex pathogens strongly implicated in periodontal disease. In contrast, *P. gingivalis* demonstrated a non-significant and highly heterogeneous response across participants, with comparable proportions showing decreases and increases over time. This variability indicates that *P. gingivalis* may be less responsive to dietary modulation alone, or may require additional host- or behaviour-related factors to be consistently affected. Taken together, these findings indicate a partial and species-specific modulation of red complex pathogens, rather than a uniform favourable shift of the red complex as a whole. Importantly, the consistent reductions observed for *T. forsythia* and *T. denticola*—two species strongly implicated in periodontal inflammation and in the pathophysiology of NCDs such as cardiovascular diseases, diabetes, cancers, and rheumatoid arthritis—support the interpretation of a reduction in specific microbiological risk factors shared with systemic conditions [2,13,32].

The strongest effects were observed at T1, indicating that the most pronounced and consistent microbiological changes occurred early during the intervention, suggesting a rapid temporal association with the dietary modification. These early changes observed could reflect an ‘ecological shock’ induced by the reduction of meat intake and the introduction of einkorn which brings some fibres, leading to an early disruption of the interdental biofilm. In contrast, changes observed at T2 were more heterogeneous and only partially sustained, and subsequent microbial resilience and secondary ecological adaptations likely explain the attenuation of effects at three months, rather than a sustained global reduction of interdental dysbiosis [30,31].

Importantly, several orange complex species exhibited changes in the opposite direction to those observed for red complex pathogens, and these shifts constitute clinically and ecologically relevant findings rather than secondary phenomena. Overall, the response of orange complex species was heterogeneous and dynamic, complicating a simplified interpretation of reduced dysbiosis. *P. intermedia* showed a significant but transient increase at T1, while *C. rectus* increased at T2. These increases likely reflect active ecological responses within the interdental biofilm rather than passive fluctuations, as both species are known to rapidly exploit ecological niches created by changes in competing taxa and resource availability. In this context, the reduction of selected red complex pathogens may have facilitated ecological substitution processes, particularly for metabolically flexible species such as *P. intermedia* [30]. In parallel, *P. micra* decreased progressively and reached statistical significance at T2, consistent with its role in gingival inflammation and its sensitivity to fibre- and phytosterol-enriched environments with anti-inflammatory properties [31]. By contrast, *F. nucleatum* remained stable over time, while the increase of *C. rectus* at T2 underscores their ecological resilience and their role as bridging species in biofilm architecture [32].

All together, these findings suggest that adherence to an einkorn-enriched PHD may be associated with a complex ecological rebalancing process, rather than acting as a simple suppressive measure of the microbiota: reducing major red complex pathogens, inducing a durable decrease in some inflammatory indicators (*P. micra*), while also allowing transient increases in selected orange complex species. This dynamic behaviour illustrates the ecological plasticity of the interdental microbiome and emphasises the importance of interpreting dietary effects as modulatory rather than uniformly suppressive [30,31,34].

In parallel, periodontal parameters improved over the course of the intervention, with PD and CAL decreasing by nearly 50% in three months, with gingivitis and BoP scores dropping by more than two-thirds. These improvements are comparable to those achieved in interventional trials evaluating plant-based diets in systemic contexts. Pappe et al. (2025) reported that a whole-food plant-based diet improved periodontal health in patients with cardiometabolic risk, although their population differed in age and systemic profile [15]. In our trial, similar improvements are observed in otherwise healthy adults, suggesting that diet-driven periodontal benefits could be generalised beyond high-risk groups. However, these findings should be interpreted with caution, as the uncontrolled design, baseline periodontal status of participants, and potential non-specific effects related to study participation may have contributed to the observed changes. Observational studies in vegetarians and vegans also suggested lower gingival bleeding prevalence but these were prone to confounding [35–37].

Exploratory endpoints also suggest digestive benefits. Stool frequency normalised among participants who initially reported fewer than one stool per day. This finding is consistent with randomised trials showing that wholegrain-rich diets improve bowel function [38] and that resistant starch increases stool frequency and softens consistency [39]. At three months, 56% of participants reported exactly one bowel movement per day, a proportion closely matching European population cohorts, where once-daily stools is the most common pattern. Other digestive symptoms such as bloating, pain, and heaviness showed no significant changes, similarly to results from Wu et al. (2025), who found that global diet quality was associated with lower risk of diarrhoea but had weaker associations with nonspecific symptoms such as bloating [40]. By contrast, postprandial drowsiness decreases significantly in our trial, consistent with prior evidence that wholegrain and legume intake stabilises glycemic response and reduces postprandial fatigue [41].

Finally, self-reported quality of life improves modestly but significantly, with SF36 scores rising from 77 at baseline to 82 at three months, in a context of already high baseline scores suggesting a ceiling effect and limited clinical interpretability. Importantly, baseline values are indeed comparable to recent European normative datasets. In the updated Hungarian norms (2025), mean scores for the eight SF-36 domains in the adult population ranged from 65 to 87 [42], while French normative data in young adults reported median scores typically between 70 and 90 across domains [43]. This indicates that participants began the trial with quality of life levels consistent with general population, leaving limited room for improvement. Nevertheless, a statistically significant increase was observed over the course of the intervention. Despite this ceiling effect, these findings suggest that dietary interventions may enhance well-being even in healthy adults with initially favourable profiles.

The strengths of this study lie in its novelty and integrative design, combining clinical, microbiological, and subjective endpoints within a One Health perspective. However, several limitations must be acknowledged. First, this study was designed as an uncontrolled, single-arm longitudinal pilot and therefore does not allow causal inference; accordingly, the findings should be interpreted as hypothesis-generating and exploratory. Second, the study population was relatively small, highly selected and highly motivated, with no control or comparison group and a relatively short follow-up duration. The sample size calculation was based on effect sizes from a previous interdental hygiene intervention [28], as no comparable dietary studies were available at the time. Although the planned sample size was not fully reached due to practical constraints, the study may be underpowered for some outcomes, and the findings should be interpreted as exploratory and hypothesis-generating. Third, adherence to the dietary intervention was primarily self-reported, which may have introduced reporting bias and limits the precision with which actual dietary exposure can be quantified. In addition, several sources of potential confounding must be considered, including possible Hawthorne effects related to close follow-up and repeated assessments, regression to the mean, and broader behavioural or lifestyle changes occurring during the intervention period beyond the dietary modification itself. Furthermore, the intervention combined multiple components, including reduced meat intake, einkorn supplementation, and repeated culinary workshops, which cannot be analytically disentangled. Therefore, the observed effects should be interpreted as resulting from the overall dietary pattern rather than being attributable to einkorn alone. Fourth, limitations related to clinical outcomes and mediation analyses should be considered. While necessary for standardisation, the exclusion of participants routinely practicing interdental hygiene or using mouthrinses may have favoured the inclusion of individuals more likely to show periodontal improvement during follow-up due to increased attention or behavioural changes. Moreover, interdental biofilm sampling involved repeated mechanical disturbance of interdental sites, which represented a theoretical source of confounding. While a transient mechanical contribution related to interdental biofilm disruption cannot be entirely excluded, available randomised controlled evidence suggested that mechanical interdental brushing alone was unlikely to fully account for the magnitude, consistency, and multisystemic nature of the longitudinal changes observed [28,44,45]. Fifth, limitations related to clinical outcomes and mediation analyses must be acknowledged. Although periodontal clinical parameters improved over the course of the intervention, no mechanistic or mediation analyses were performed. Therefore, it is not possible to determine whether these clinical improvements were directly related to changes in interdental microbiota or to non-specific behavioural or contextual factors. Sixth, limitations relate to the interpretation of microbiological findings. In the present study, dysbiosis was operationally defined as the quantitative burden and longitudinal changes of selected periodontal pathogens from the red and orange complexes assessed by targeted qPCR, a deliberate choice that prioritised sensitive absolute quantification and longitudinal comparability over community-level sequencing approaches (e.g. 16S rRNA gene sequencing or shotgun metagenomics). This definition does not capture community-level microbial diversity, global ecological structure, or functional interactions within the interdental microbiome. Importantly, changes in bacterial abundance should not be equated with changes in pathogenicity or disease risk, as no functional or virulence-related or host-response pathways analyses were performed in this study. Consequently, the microbiological findings should be interpreted as reflecting changes in pathogen burden rather than a comprehensive characterisation of community-wide dysbiosis. Future studies incorporating community-level sequencing approaches or composite indices such as the Subgingival Microbial Dysbiosis Index (SMDI) [46], including yellow and green complex species as proposed by Chen et al. (2022), would allow a more comprehensive ecological assessment of interdental microbial shifts. These factors preclude strong causal inference. Nevertheless, in comparison with existing studies, our results provide exploratory interventional data suggesting that sustainable dietary choices can simultaneously promote oral microbial balance, periodontal health, and digestive comfort.

Importantly, the microbiological shifts observed in this study should not be interpreted as evidence of reduced pathogenicity or disease risk. Changes in bacterial abundance do not equate to changes in biological activity or virulence, as no mechanistic, functional, or host-response pathways were investigated. Accordingly, the present findings should be considered hypothesis-generating and intended to inform future controlled studies rather than to demonstrate preventive effects.

Despite these limitations, this proof-of-concept study provides exploratory evidence that adherence to an einkorn-enriched PHD is associated with measurable changes in interdental microbiota, periodontal parameters, and selected self-reported health outcomes. These findings support the biological plausibility that sustainable dietary patterns may influence oral microbial ecosystems and periodontal inflammation, pathways that are increasingly recognised as being involved in the broader pathophysiology of non-communicable diseases. Taken together, the present results should be interpreted as hypothesis-generating, reinforcing the relevance of integrating sustainable nutrition into oral health research within a One Health framework, and underscoring the need for larger, controlled, and longer-term studies to determine whether the observed oral and microbiological changes are relevant to systemic outcomes related to non-communicable diseases.

## Conclusion

This uncontrolled pilot clinical trial suggests that adherence to an einkorn-enriched PHD was associated with early and partially sustained shifts in selected interdental pathogens, with the strongest microbiological changes observed at one month and more heterogeneous patterns at three months, as well as favourable longitudinal changes in periodontal parameters and modest changes in digestive comfort and quality of life. These exploratory findings support a conceptual link between sustainable dietary choices, oral and systemic health within a One Health framework, linking human well-being, nutrition, and planetary sustainability. Larger controlled studies are warranted to confirm these observations and to clarify their potential relevance for broader preventive strategies.

## Supplementary Material

Supplementary material BIOQUALIM clinical study STROBE.

Supplementary material BIOQUALIM_supplementary_JOM.

## Data Availability

Deidentified data are available upon request to the corresponding author.
