# Supplementary material for: Planetary health diet with einkorn as a potential preventive strategy to improve interdental microbiota, oral health and quality of life: a pilot clinical trial
Source: J Oral Microbiol. 2026 Feb 9;18(1):2626138. doi: 10.1080/20002297.2026.2626138 (PMC12888355; doi:10.1080/20002297.2026.2626138)
Supplement: Supplementary material — BIOQUALIM_supplementary_JOM. [file ZJOM_A_2626138_SM5509.docx]

**Supplementary files**

**File 1.** Questionnaire on eating habits and digestive discomfort

**Eating habits**

In a typical week with 7 lunches and 7 dinners, how often do you eat:

Red meat (beef, pork, veal, mutton, lamb, goat, horse, wild boar, venison, etc.)

□ 0 times □1 to 2 times □ 3 to 4 times □ 5 to 7 times □ more than 7 times

White meat (chicken, turkey, rabbit, etc.)

□ 0 times □1 to 2 times □ 3 to 4 times □ 5 to 7 times □ more than 7 times

Cold cuts and processed meat (cooked or raw ham, sausages and merguez, blood sausage, andouillette, salami, bacon, corned beef, beef jerky, pâté, rillettes, kebabs, meatballs, etc.)

□ 0 times □1 to 2 times □ 3 to 4 times □ 5 to 7 times □ more than 7 times

Eggs (eaten as is or in preparations such as cakes, etc.)

□ 0 times □1 to 2 times □ 3 to 4 times □ 5 to 7 times □ more than 7 times

Fish, seafood, and shellfish (salmon, tuna, mackerel, sardines, herring, cod, sea bass, sea bream, mussels, shrimp, oysters, etc.)

□ 0 times □1 to 2 times □ 3 to 4 times □ 5 to 7 times □ more than 7 times

Whole grains or bulgur wheat from cereals other than einkorn wheat (wheat, spelt, oats, barley, rye, sorghum, millet) and/or other non-cereal species (quinoa, buckwheat)

□ 0 times □1 to 2 times □ 3 to 4 times □ 5 to 7 times □ more than 7 times

Legumes (lentils, chickpeas, split peas, broad beans, soybeans, dried beans, dried peas, lupins, tofu, tempeh, textured soy protein, soy steak)

□ 0 times □1 to 2 times □ 3 to 4 times □ 5 to 7 times □ more than 7 times

Have you used other products derived from non-hybridized einkorn or emmer wheat (before/since the start of the study)?

□ Yes, by eliminating wheat □ Yes, concurrently with my wheat consumption □ No

**Digestive comfort**

How many times do you have a bowel movement (on average):

□ 3 times or more/day □ 2 times/day □ 1 time/day □ 3 to 4 times/week □ less than 3 times/week

Do you feel bloated after or between meals?

□ Never □ Rarely □ Sometimes □ Often □ Very often

Do you experience digestive pain after or between meals?

□ Never □ Rarely □ Sometimes □ Often □ Very often

Do you feel heavy after meals?

□ Never □ Rarely □ Sometimes □ Often □ Very often

Do you feel full 2 hours after meals?

□ Never □ Rarely □ Sometimes □ Often □ Very often

Do you feel sleepy after meals?

□ Never □ Rarely □ Sometimes □ Often □ Very often

Did you use any other products derived from einkorn or emmer or spelt during the study?

□ Never □ Rarely □ Sometimes □ Often □ Very often

**Figure 1.** Flowchart of the study, a single-arm pilot intervention without control group

Screened for eligibility

(n = 200)

**Identification**

Not eligible: n = 168

- refused to participate (n = 160)

- refused to provide written informed consent (n = 3)

- not met inclusion criteria (n = 5)

Included

(n = 27)

Excluded:

- not presented (n = 2)

**Inclusion**

Completed 1-month follow-up

(n = 25)

**Follow up**

Completed 3-month follow-up

(n = 25)

**Analysis**

Analyzed

(n = 25)

**Table 1.** Reported eating habits at baseline and at the end of the study.

*Test de Bowker

|  | **T0 N=25**  **n (%)** | **T2 N=25**  **n (%)** | **p-value*** |
| --- | --- | --- | --- |
| **Weekly consumption habits** |  |  |  |
| Red meat |  |  | **0.0045** |
| - 0 time | 1 (4.0%) | 2 (8.0%) |  |
| - 1 to 2 times | 13 (52.0%) | 16 (64.0%) |  |
| - 3 to 4 times | 8 (32.0%) | 7 (28.0%) |  |
| - 5 to 7+ times | 3 (12.0%) | 0 |  |
| White meat |  |  | **0.0075** |
| - 0 time | 2 (8.0%) | 2 (8.0%) |  |
| - 1 to 2 times | 12 (48.0%) | 15 (60.0%) |  |
| - 3 to 4 times | 7 (28.0%) | 6 (24.0%) |  |
| - 5 to 7+ times | 4 (16.0%) | 2 (8.0%) |  |
| Cured meat |  |  | **0.033** |
| - 0 time | 3 (12.0%) | 6 (24.0%) |  |
| - 1 to 2 times | 16 (64.0%) | 15 (60.0%) |  |
| - 3 to 4 times | 3 (12.0%) | 4 (16.0%) |  |
| - 5 to 7+ times | 3 (12.0%) | 0 |  |
| Eggs |  |  | 0.6547 |
| - 0 time | 0 | 0 |  |
| - 1 to 2 times | 9 (36.0%) | 9 (36.0%) |  |
| - 3 to 4 times | 11 (44.0%) | 9 (36.0%) |  |
| - 5 to 7+ times | 5 (20.0%) | 7 (28.0%) |  |
| Fish, seafood and crustaceans |  |  | **0.0046** |
| - 0 time | 4 (16.0%) | 3 (12.0%) |  |
| - 1 to 2 times | 15 (60.0%) | 18 (72.0%) |  |
| - 3 to 4 times | 4 (16.0%) | 3 (12.0%) |  |
| - 5 to 7+ times | 2 (8.0%) | 1 (4.0%) |  |
| Whole grains or bulgur of cereals other than einkorn |  |  | 0.1572 |
| - 0 time | 7 (28.0%) | 6 (24.0%) |  |
| - 1 to 2 times | 12 (18.0%) | 7 (28.0%) |  |
| - 3 to 4 times | 3 (12.0%) | 4 (16.0%) |  |
| - 5 to 7+ times | 3 (12.0%) | 6 (32.0%) |  |
| Leguminous plants |  |  | **0.0075** |
| - 0 time | 5 (20.0%) | 2 (8.0%) |  |
| - 1 to 2 times | 12 (48.0%) | 12 (48.0%) |  |
| - 3 to 4 times | 8 (32.0%) | 9 (36.0%) |  |
| - 5 to 7+ times | 0 | 2 (8.0%) |  |

**Table 2. Difference of log10-transformed bacterial counts at baseline (T0) and 1 month (T1) compared to 3 months (T2).**

*Pg: Porphyromonas gingivalis, Tf: Tanerella forsythia; Td: Treponema denticola; Fn: Fusobacterium nucleatum; Pi: Prevotella intermedia; Prevotella micra; Cr: Campylobacter rectus.*
